# Some See It, Some Don’t: Exploring the Relation between Inattentional Blindness and Personality Factors

**DOI:** 10.1371/journal.pone.0128158

**Published:** 2015-05-26

**Authors:** Carina Kreitz, Robert Schnuerch, Henning Gibbons, Daniel Memmert

**Affiliations:** 1 Institute of Cognitive and Team/Racket Sport Research, German Sport University Cologne, Cologne, Germany; 2 Department of Psychology, University of Bonn, Bonn, Germany; University of Akron, UNITED STATES

## Abstract

Human awareness is highly limited, which is vividly demonstrated by the phenomenon that unexpected objects go unnoticed when attention is focused elsewhere (inattentional blindness). Typically, some people fail to notice unexpected objects while others detect them instantaneously. Whether this pattern reflects stable individual differences is unclear to date. In particular, hardly anything is known about the influence of personality on the likelihood of inattentional blindness. To fill this empirical gap, we examined the role of multiple personality factors, namely the Big Five, BIS/BAS, absorption, achievement motivation, and schizotypy, in these failures of awareness. In a large-scale sample (N = 554), susceptibility to inattentional blindness was associated with a low level of openness to experience and marginally with a low level of achievement motivation. However, in a multiple regression analysis, only openness emerged as an independent, negative predictor. This suggests that the general tendency to be open to experience extends to the domain of perception. Our results complement earlier work on the possible link between inattentional blindness and personality by demonstrating, for the first time, that failures to consciously perceive unexpected objects reflect individual differences on a fundamental dimension of personality.

## Introduction

One of the most essential functions of our cognitive system, the ability to selectively prioritize certain sensations, sometimes leads us to inadvertently exclude other sensory information from awareness: We often miss an object right in front of our eyes if it occurs unexpectedly and our attention is otherwise engaged. This phenomenon has been labeled inattentional blindness [1], and it occurs in diverse and potentially critical situations. For example, when pilots focus on information projected onto the windshield during landing, they sometimes completely overlook other airplanes on the runway [2]. Also, radiologists looking for lung nodules in CT scans failed to notice much larger, yet unexpected alterations in the image [3] (see also[4]).

Considering the potential harm and impact of such oversight, researchers have begun to unravel the underlying mechanisms. In particular, previous research has explored to what extent such failures of awareness depend on contextual factors. For example, several features of the unexpected object itself influence the probability of inattentional blindness: its size [1], its color [5], its semantic content (e.g., [6,7]), and its distance from the attentional focus [8,9]. Also, the likelihood of inattentional blindness is affected by the cognitive demands of the primary task [10,11] and the observer's current attentional goals [12–14]. Thus, the contribution of situational factors has been investigated in considerable depth and is well-established. Somewhat surprisingly, it is still unclear whether the susceptibility to inattentional blindness also depends on stable individual differences.

The typical finding in inattentional blindness studies is that some people fail to notice an unexpected object while others detect it instantaneously. In this context, researchers rarely address whether this pattern reflects individual differences in the ability to notice unexpected objects or there is the same fixed probability to notice the object for everyone. That is, are the people who noticed the object in any way systematically different from those who did not? Driven by the idea that it might be easier to detect an unexpectedly appearing object if one's cognitive resources are not exhausted by the primary task, previous studies have mainly focused on participants' cognitive capacity. The results are, however, ambiguous, with some studies showing a direct link between noticing unexpected objects and working memory capacity [15–17], while others found only a restricted relation [6,18] or even no relation at all [19].

The role of individual differences regarding personality is even less well-studied. To the best of our knowledge, there are only two studies to date investigating whether inattentional blindness is related to personality factors in adults [20,21]. A related third study showed a negative association between inattentional blindness and creative thinking in seven-year-old children [22]. We did, however, not follow up on this specific finding for two reasons. First, whether creativity qualifies as a personality trait (rather than a cognitive ability) is subject to debate [23,24]. Second, creativity is particularly difficult to assess, even in laboratory settings [25]. Thus, it seemed hardly feasible to reliably measure it in an online scenario.

Based on findings that both depression and anxiety are linked to attentional control deficits, Bredemeier et al. [20] tested whether individual differences in emotional distress influenced the probability of inattentional blindness. Their findings did not support a direct association between the susceptibility to miss an unexpected object and self-reported levels of trait negative affect, anhedonic depression, anxious arousal, or worry. Richards et al. [21] tested whether paranormal belief and absorption, the trait tendency to become absorbed in a momentary experience, is negatively associated with noticing unexpected objects. The authors argued that absorption implies inhibition of stimuli outside the attentional focus and should therefore be related to inattentional blindness. In line with this prediction, participants who scored higher on the absorption scale and the paranormal-belief scale were more likely to be inattentionally blind. The relation between paranormal belief and inattentional blindness was indirect, as it was mediated by absorption.

The results of Richards et al. [21] are the first to demonstrate a link between the susceptibility to inattentional blindness and a stable personality trait. The findings are indeed convincing, given their high face validity and the fact that the authors were able to replicate their findings in a separate sample within the same study. They illustrate that the role of personality in inattentional blindness is a relevant and promising avenue of research, yet it is still sparse and clearly limited. Clarifying whether, and to what extent, personality contributes to such blatant failures of awareness could enhance our general understanding of the underlying mechanisms of the phenomenon. It would complement our perspective on inattentional blindness, which is predominantly shaped by cognitive and situational predictors (see, e.g., [13,16]). Thus, in the present study, we further explored the importance of individual differences in personality traits for inattentional blindness. We aimed at replicating the findings of Richards et al. [21] regarding absorption and broadening them, as well as the findings of Bredemeier et al. [20], to a set of more classical personality traits.

### The Present Study

In the present study, we applied a twofold approach to the investigation of personality in inattentional blindness. As a first, theory-driven step, we assessed a set of personality traits that seem promising candidates for a relationship with inattentional blindness: (1) absorption, (2) the Big Five personality factor openness to experience, (3) schizotypy, and (4) achievement motivation. In a second, more exploratory step, we additionally measured fundamental and classical dimensions of personality that have not yet been linked to individual differences in inattentional blindness, namely (5) behavioral inhibition vs. activation and (6) the remaining Big Five personality factors.

1. The susceptibility to become absorbed in a momentary experience is a stable personality trait that continuously differs between individuals. It can be measured with the Tellegen Absorption Scale (TAS; [26]) and reflects the tendency to become engaged in highly focused attentional states in which all unattended objects or events are shut out [26]. The possible connection to inattentional blindness seems straightforward as participants are engaged in a demanding primary task when suddenly an unexpected, and thus unattended, object appears. And indeed, Richards and colleagues [21] demonstrated that participants scoring higher on the TAS are more likely to miss an unexpected object during a dynamic and sustained inattentional blindness task (i.e., a task in which the unexpected objects is in motion and present for several seconds; see [27]). We included *absorption* in our test battery in order to replicate this finding and see if it holds with a static inattentional blindness task (i.e., a task in which the unexpected object is stationary and present for only 200 ms; see [1]).
2. One of the fundamental dimensions of personality is *openness* [28]. It is typically measured by assessing self-reported characteristics such as one’s knowledge of music, art, and literature, one’s imaginativeness, or one’s resourcefulness [29]. In addition to describing this general receptiveness to many varieties of new experiences, openness might also subsume a more perceptual capability by reflecting a “fluid and permeable structure of consciousness” ([30], p. 251). Thus, it is conceivable that individuals who are open to new experiences might also be more receptive (in a perceptual sense) to unexpected objects or events. This notion is indirectly backed by empirical evidence: Creative individuals score higher on the openness scale [31], and they are also more likely to detect unexpected objects in an inattentional blindness paradigm [22]. Openness might therefore also be connected to inattentional blindness. We hypothesized that a high openness to experience is accompanied by a reduced proneness to inattentional blindness.
3. Psychotic proneness, the tendency to detach from reality, is also labeled *schizotypy* and can be measured as a continuum in healthy individuals [32]. This specific personality trait is of potential interest for individual differences in inattentional blindness because it has repeatedly been shown to be related to attentional capabilities [33,34]. Specifically, schizotypy is related to the processing of irrelevant information: Individuals scoring high on schizotypy show less latent inhibition, which is a retardation of learning following preexposure to a formerly irrelevant stimulus [35,36]. Thus, individuals who are prone to psychosis do not adapt as strongly to irrelevant stimuli as individuals who are less prone to psychosis. As these well-established findings indicate that highly schizotypic individuals do not filter sensory input effectively, we hypothesized that they likewise might be more responsive to unexpected stimulation. Thus, they should be less prone to inattentional blindness.
4. Richards et al. [16] addressed the possibility that motivational differences between observers might predict differences in inattentional blindness. They operationalized motivation as accuracy and speed in the currently performed primary task and did not find evidence for a relation to inattentional blindness. Memmert, Unkelbach, and Ganns [37] reported that inattentional blindness decreased when there was a match between a person's general motivational orientation (promotion vs. prevention; see [38,39]) and the demands of the experimental situation. Thus, these findings provide a first hint that individual differences in motivation modulate susceptibility to inattentional blindness. However, while both Memmert et al. [37] and Richards et al. [16] focused on measures that capture both chronic and situational aspects of motivation, we wanted to register it as a stable personality trait. Therefore, we assessed participants’ *achievement motivation* [40], that is, the effort an individual habitually makes to reach certain performance goals, for example, academic or job-related goals. We hypothesized that a higher motivation might result in a lower probability of inattentional blindness, possibly because more attentional resources are summoned during the experimental procedure.
5. For exploratory purposes, we additionally included all Big Five personality traits (see [41,42]). In addition to openness (see above), the Big Five comprise the personality traits *conscientiousness* (the tendency to be organized, efficient, and dependable), *extraversion* (the tendency to be outgoing and energetic), *agreeableness* (the tendency to be friendly and compassionate), and *neuroticism* (the tendency to be sensitive and nervous). Together, the Big Five represent the most fundamental dimensions of each individual’s personality structure [43].
6. Another fundamental aspect of personality, largely based on biopsychological aspects of individual differences, is the degree by which individuals are driven by reward and/or anxiety [44,45]. According to Gray [44,45], this corresponds to the strength of their *behavioral inhibition system* (BIS; an aversive motivational system related to anxiety) and their *behavioral activation system* (BAS; an appetitive motivational system related to reward). Individual differences in these systems presumably reflect variations in general cortical activity [46] and have been linked to fundamental cognitive functioning [47]. Separate BIS and BAS scales measure individual differences in behavior and experience on these two dimensions [48]. As BIS and BAS constitute another fundamental classification of individual differences in personality, we included them for exploratory purposes in addition to the Big Five.

## Method

### Ethics statement

The reported study was conducted as part of a larger research project on individual differences in inattentional blindness, which was reviewed and approved by the ethics committee of the German Sport University Cologne. Participants declared their consent online and could only then proceed with the test and questionnaires. They were debriefed afterwards.

### Participants

Data were obtained in an online study. We distributed the hyperlink to the study via Facebook and diverse bulletin boards. Mostly, we targeted Facebook groups of universities and major German cities. By taking part in the study, participants had the chance to win one of three Amazon gift certificates (50€ each). Overall, 3206 people responded to our call and started working on the tasks and questionnaires. Of those, 1104 actually completed the whole study. Because we can act on the assumption that the conditions in which participants completed the study were very diverse and, on top of that, mostly inaccessible to us, we predefined strict exclusion criteria. Participants were excluded from analysis if (1) they did not see both inattentional blindness videos because of technical problems, (2) they did not notice the unexpected object in the control condition (full-attention trial), (3) they knew or assumed beforehand that the task was actually about noticing an unexpected object, (4) they did not perform perfectly on the primary task in the inattentional blindness video, or (5) they reported uncorrected impaired vision. Based on these criteria, 550 participants were excluded, leaving 554 for analysis. The age of the analyzed participants ranged from 18 to 59 years (*M* = 26.0 years, *SD* = 6.5 years) and 414 (74.7%) of them were women.

### Materials and Procedure

The study was run online (https://www.soscisurvey.de). First, the inattentional blindness task was presented. The task consisted of two videos (one containing the critical event and one serving as control under full attention) and subsequent questions about the unexpected object. Also, we enquired participants' anticipation of the additional object and their general knowledge of inattention blindness. Thereafter, the questionnaires were presented in the same order as described below. After completion of all inventories, we collected demographics and information about the technical device on which participants had completed the study. Also, we asked if both videos had played without problems. Finally, participants were debriefed and could leave their e-mail address if they wanted to participate in the drawing of the three gift certificates.

#### Inattentional Blindness Task

As inattentional blindness task, we employed a static task in which an unexpected object was presented for 200 ms alongside the primary-task stimulus. This task was adapted from procedures presented by Mack and Rock [1] and has been used in a similar form by Kreitz, Schnuerch, Furley, Gibbons, and Memmert [49]. Specifically, participants were asked to perform a lexical-decision task. They watched a video in which letter strings, consisting of four black letters, were presented for 200 ms on a white background. Each letter string was preceded by a small fixation cross presented for 2000 ms. The letter string either formed a proper German word or was a meaningless non-word. Participants were instructed to watch carefully and take a silent count of the proper words. Participants saw 12 letter strings (of which five were proper words) before an additional and unexpected object was presented in a 13th trial. The unexpected object was a gray square (12 x 12 pixels; RGB: 232,232,232) that was presented alongside a non-word string (“PLIN”) for the entire 200 ms. The square appeared slightly above and to the left of the letter string with an approximate distance from the center of 105 pixels. Immediately after this critical trial, participants typed in the total number of proper words they had counted and were then asked whether they had seen anything additional that had not been part of the task. Irrespective of their response, participants were asked where the additional object had been presented (upper right, lower right, lower left, upper left), which color (red, green, yellow, blue, gray), and which shape (rectangle, square, triangle, diamond, cross, cross rotated by 45°) it had. They were told to guess in case they had not noticed anything. After these questions, participants again fixated on the center of the display and watched another video, but did not have to perform the lexical decision any more (full attention). The full-attention trial consisted of only one trial: Following a fixation cross (2000 ms), the additional square was again presented next to a non-word string (same position as at its first appearance). The questions concerning the additional object were exactly the same as those presented before.

#### Big Five Inventory (BFI)

The BFI measures five basic personality traits via 44 items [41]. We used the German version by Lang, Lüdtke, and Asendorpf [29]. Extraversion is measured by eight items (three inverted items), agreeableness is measured by nine items (four inverted items), conscientiousness is measured by nine items (four inverted items), neuroticism is measured by eight items (three inverted items), and openness is measured by ten items (two inverted items). Participants are asked to rate how well certain statements apply to them on a scale from 0 (*does not apply at all*) to 4 (*applies very well*). The items from the different scales are listed in an intertwined order. Scores are calculated by averaging the responses of all items for each scale (possible scores range from 0 to 4). The German scales have been found to be reliable and valid [29].

#### Behavioral Inhibition and Behavioral Activation Scales (BIS/BAS)

We used the BIS/BAS scales [48] to measure a more biopsychological aspect of personality, namely the two motivational systems of inhibition and activation [44,45]. We employed the German version [50] of the original questionnaire developed by Carver and White [48]. The questionnaire consists of 24 items of which seven measure BIS (two items inverted), 13 measure BAS (no inversion), and four are dummy items that are not integrated into the scales. Responses are given on a 4-point Likert scale ranging from *does not apply at all to me* (1) to *completely applies to me* (4). Scale scores are calculated by averaging the item scores and thus range from 1 to 4. The German version has acceptable psychometric properties [50]. However, a distinction into three different BAS subscales, as was suggested by Carver and White [48], cannot be supported with the German version and is disadvised by the authors [50].

#### Achievement Motivation Scale (LMI)

To measure individual differences in achievement motivation, we used the German version of the achievement motivation scale (Leistungsmotivationsinventar; [40]). The long version comprises 170 items and measures 17 subscales. We used a short version consisting of 30 items measuring achievement motivation as a single dimension. The short scale has been shown to be reliable and to correlate highly with the overall score of the long version [40]. Participants answered the 30 items on a 7-point Likert scale which ranges from *does not apply at all* (1) to *completely applies* (7). One item is inversely coded. The total score is calculated by summing all the item scores and thus ranges from 30 to 210.

#### Tellegen Absorption Scale (TAS)

The TAS is a 34-item scale that measures absorption, an individual's susceptibility to become engrossed in highly focused attentional states [26]. In contrast to this original scale, the German version [51] does not collect binary *true*/*false* responses, but measures responses on a 5-point Likert scale ranging from *does not apply* (0) to *fully applies* (4). The TAS does not contain reversely coded items. The total score is obtained by summing the item scores (scores range from 0 to 136). The German version shows sufficiently high reliabilities [51].

#### Oxford-Liverpool Inventory of Feelings and Experiences (O-LIFE)

The O-LIFE measures schizotypy, which is psychotic proneness [32]. We used the German short version by Grant et al. [52], which comprises 43 items, of which eight are reversely coded. Each item poses a question demanding a *yes* or *no* answer. The test score reflects the number of positive answers and thus ranges from 0 to 43. The O-LIFE measures four subscales: unusual experiences (12 items), cognitive disorganisation (11 items), introvertive anhedonia (10 items), and impulsive nonconformity (10 items). We did, however, concentrate on the overall scale in the present study as we did not have separate hypotheses regarding the subscales. The German translation of the short version of the O-LIFE is both highly reliable and consistent with the original measure [52].

## Results

Participants were considered to have missed the unexpected object if they did not report noticing it or claimed to have seen something but could not define at least two of the following three features of the unexpected object: position, color, shape. Since we only included data from participants who noticed the additional gray square in the control condition (full attention) and correctly identified two of its three features, missing the *critical* unexpected object cannot be attributed to unwanted physical or technical difficulties (such as basal visual problems, a poor contrast, or other factors related to the particular technical device that the participants used to perform the study). All statistical analyses conducted were two-tailed. Correlations are reported with 95% confidence intervals in square brackets. As a standardized measure of effect size for group differences, risk ratios with 95% confidence intervals in square brackets are reported.

### Power Analysis

Prior to our analyses, we ascertained whether the sample size was sufficient to achieve high statistical power. This is particularly important when null effects are reported, as power describes the probability that a test actually rejects an incorrect null hypothesis [53,54]. It can be interpreted as an index of the sensitivity of a test to detect an effect of a certain size [55]. To test this for the present data, we performed a post-hoc power analysis using the software G*power 3 [54]. Given our final sample size (*N* = 554) and our alpha level of. 05, this analysis revealed that the power (1 – β) in our analyses was. 95 even for small-to-medium effects. That is, we were able to detect point-biserial correlations as small as *r* = .15 with a probability of. 95. Thus, the statistical approach of the present study can be considered powerful, which makes it highly unlikely that any null effects are simply due to a lack of statistical sensitivity.

### Technical Conditions

Neither screen type nor absolute screen size influenced noticing rates (*r* = -.04 [-.12,. 04], *p* = .40 and *r* = .04 [-.05,. 12], *p* = .39, respectively). Also, the self-reported technical up-to-dateness was unrelated to noticing (*r* = .06 [-.02,. 14], *p* = .18). Thus, it did not matter at which screen or device the inattentional blindness video was viewed. Consequently, we did not add those parameters to the list of exclusion criteria and did not further consider them in our analysis. Importantly, these results indicate that conducting an inattentional blindness experiment as an online study is feasible and largely unconfounded by specific and individual technical conditions.

### Main Analyses

Descriptive values of the personality measures are shown in Table 1. All scales showed substantial variation within our sample and, thus, fulfilled an important requirement to exhibit relationships with other measures. Reliability of the different scales, measured as internal consistency, ranged from sufficient to excellent (Cronbach’s alpha. 72 to. 94). Interrelations between the predictors (i.e., scales and demographic variables) are shown in Table 2. A total of 351 out of the 554 participants (63.4%) did not notice the unexpected shape. Thus, we successfully generated inattentional blindness without any floor or ceiling effects that might conceal potential relationships with the personality measures.

**Table 1:** Descriptive data and consistency of the personality scale.

	Items	*M*	*SD*	α
Big Five—Openness	10	2.66	0.62	.81
Big Five—Conscientiousness	9	2.44	0.61	.81
Big Five—Extraversion	8	2.35	0.76	.89
Big Five—Agreeableness	9	2.54	0.54	.72
Big Five—Neuroticism	9	2.01	0.74	.86
BIS/BAS—BIS	7	3.05	0.55	.82
BIS/BAS—BAS	13	3.10	0.36	.75
Achievement Motivation (LMI)	30	140.90	26.95	.94
Tellegen Absorption Scale (TAS)	34	68.04	26.26	.94
Schizotypy (O-LIFE)	43	16.82	6.35	.79

*Note*. *SD* = standard deviation, α = internal consistency measured by Cronbach’s alpha

**Table 2:** Correlations (Pearson’s r) among the predictor variables.

	1	2	3	4	5	6	7	8	9	10	11	12
(1) Openness to experience	—-	.02	.14	.13	-.07	.49	.29	-.04	.24	.12	.00	.06
(2) Conscientiousness	[-.06,. 10]	—-	.17	.22	-.15	-.12	.44	-.06	.19	-.35	-.06	.01
(3) Extraversion	[.06,. 22]	[.09,. 25]	—-	.07	-.26	.03	.31	-.27	.47	-.24	-.11	.05
(4) Agreeableness	[.05,. 21]	[.14,. 30]	[-.01,. 15]	—-	-.25	.08	.03	.02	.04	-.20	-.06	.06
(5) Neuroticism	[-.15,. 01]	[-.23,-.07]	[-.34,-.18]	[-.33,-.17]	—-	.10	-.23	.73	-.08	.45	-.28	-.14
(6) Absorption	[.42,. 55]	[-.20,-.04]	[-.05,. 11]	[.00,. 16]	[.02,. 18]	—-	.07	.16	.26	.47	-.09	.01
(7) Achievement Motivation	[.21,. 36]	[.37,. 51]	[.23,. 38]	[-.05,. 11]	[-.31,-.15]	[-.01,. 15]	—-	-.26	.47	-.14	.14	-.01
(8) BIS	[-.12,. 04]	[-.14,. 02]	[-.35,-.19]	[-.06,. 10]	[.69,. 77]	[.08,. 24]	[-.34,-.18]	—-	-.02	.37	-.35	-.12
(9) BAS	[.16,. 32]	[.11,. 27]	[.40,. 53]	[-.04,. 12]	[-.16,. 00]	[.18,. 34]	[.40,. 53]	[-.10,. 06]	—-	.03	-.06	-.07
(10) Schizotypy	[.04,. 20]	[-.42,-.28]	[-.32,-.16]	[-.28,. 12]	[.38,. 51]	[.40,. 53]	[-.22,-.06]	[.30,. 44]	[-.05,. 11]	—-	-.11	-.07
(11) Gender	[-.18,. 18]	[-.14,. 02]	[-.19,-.03]	[-.14,. 02]	[-.36,-.20]	[-.17,-.01]	[.06,. 22]	[-.42,-.28]	[-.14,. 02]	[-.19,-.03]	—-	.03
(12) Age	[-.02,. 14]	[-.07,. 09]	[-.03,. 13]	[-.02,. 14]	[-.22,-.06]	[-.07,. 09]	[-.09,. 07]	[-.20,-.04]	[-.15,. 01]	[-.15,. 01]	[-.05,. 11]	—-

*Note*. The lower and upper bounds of the 95% confidence interval are shown in square brackets below the diagonal. Correlational values ≥ |.09| are significant at an alpha level of. 05, values ≥ |.12| are significant at an alpha level of. 01, and values ≥ |.15| are significant at an alpha level of. 001. N = 554 for all correlations

We conducted a binary logistic regression to determine which of the a-priori hypothesized personality measures (absorption, openness, schizotypy, and achievement motivation) significantly predicted noticing. The dependent variable was noticing (0 or 1 per participant), and the four personality traits were included as predictors by the enter method. As displayed in Table 3, the overall model was significant and accounted for approximately 3% of the variance in noticing of the unexpected object. Interestingly, only openness was a significant independent predictor of noticing.

**Table 3:** Results of the binary logistic regression with simultaneous entry (SE in parentheses).

Variables	*B* (*SE*)	Wald	Exp(*B*)	Exp(*B*) lower	Exp(*B*) upper	*p*
Constant	-2.34 (0.63)	14.02	0.10			< .001
Openness to experience	0.51 (0.18)	8.23	1.66	1.17	2.35	.004
Absorption	-0.01 (0.00)	2.92	0.99	0.98	1.00	.087
Achievement Motivation	0.00 (0.00)	1.46	1.00	1.00	1.01	.227
Schizotypy	0.02 (0.02)	1.51	1.02	0.99	1.05	.219
*R* ^*2*^ = .*02 (Cox & Snell)*, *R* ^*2*^ = .*03 (Nagelkern)*, *Model*: χ^*2*^ *(4) = 13*.*05*, *p* = .*011*						

*Note*. The upper and the lower bounds of the 95% confidence interval of Exp(*B*) are depicted as well.

Table 4 displays the correlations between all personality traits measured in the present study and noticing of the unexpected object, including both the theory-driven and exploratory factors. Resembling the finding of the logistic regression, a significant relationship was solely evident for openness. The effect size (r = .12 [.04,. 20]), however, has to be classified as small and thus indicates a rather limited relationship [56]. The relationship between noticing and achievement motivation was borderline significant. This effect size is also classified as small. All other personality traits were not related to noticing the unexpected object.

**Table 4:** Correlations of noticing with the personality factors.

	correlation	CI lower bound	CI upper bound	*p*
Openness to experience	.12	.04	.20	.003
Conscientiousness	.02	-.06	.10	.197
Extraversion	-.01	-.09	.07	.866
Agreeableness	-.06	-.14	.02	.197
Neuroticism	.03	-.05	.11	.552
Absorption	.01	-.07	.09	.823
Achievement Motivation	.08	-.00	.16	.057
BIS	-.03	-.11	.05	.499
BAS	-.01	-.09	.07	.745
Schizotypy	.03	-.08	.08	.536

*Note*. Displayed are point-biserial correlations and their 95% confidence intervals.

Previous research repeatedly did not find gender differences in inattentional blindness [15,16]. Also, age does not seem to affect the susceptibility to this failure of awareness in adult samples [15,16] (but see [57] for differences among children). In accordance with this, we did not find an effect of age in the present study (*r* = .07 [-.01,. 15], *p* = .12). We did, however, find a substantial effect of gender, with men noticing the unexpected object significantly more often than women (χ^2^(1) = 11.48, *p* = .001, risk ratio(men/women) = 1.49 [1.20, 1.86]). To assess whether gender possibly mediated the effect of openness on noticing, we conducted an additional regression analysis in which both openness and gender were included as predictors by the enter method. Table 5 shows that the predictive value of openness did not disappear when gender was included in the logistic regression. Rather, gender and openness were similarly strong, independent predictors of noticing an unexpected object.

**Table 5:** Results of the binary logistic regression with simultaneous entry (SE in parentheses).

Variables	*B* (*SE*)	Wald	Exp(*B*)	Exp(*B*) lower	Exp(*B*) upper	*p*
Constant	-1.91 (0.42)	20.49	0.15			< .001
Openness to experience	0.44 (0.15)	8.58	1.55	1.16	2.08	.003
Gender	0.68 (0.20)	11.51	1.98	1.33	2.93	.001
*R* ^*2*^ = .*04 (Cox & Snell)*, *R* ^*2*^ = .*05 (Nagelkern)*, *Model*: χ^*2*^ *(2) = 20*.*08*, *p <* .*001*						

*Note*. The upper and the lower bounds of the 95% confidence interval of Exp(*B*) are depicted as well.

It should be noted that all of the above analyses were performed after carefully excluding participants on the basis of a-priori criteria (see Method section). However, it might be interesting (e.g., for further online experimentation in this field) to explore whether the selection of criteria altered the general pattern of results. To test this, we re-analyzed all data without excluding any participants, as well as by selectively dropping each of the five criteria. Most importantly, the overall pattern of results stayed the same across all analyses (see S1 File for details). As could be expected, though, data were noisier, especially if no exclusion criterion was applied. Thus, in essence, a careful application of a-priori criteria for data inclusion is highly expedient, especially given the conditions of online experimentation. Moreover, it is a well-established strategy to exclude certain participants in IB studies, for example, dropping those who did not even notice the unexpected object under conditions of full attention (see, e.g., [8,49]).

## Discussion

In the attempt to disentangle the underlying mechanisms of inattentional blindness, a substantial amount of research has been conducted on the situational factors that affect the susceptibility to this failure of awareness (e.g. [8,9,58,59]). Far less research has been concerned with individual differences that might influence proneness to inattentional blindness. In particular, hardly anything is known about the contribution of personality to this phenomenon (but see [20,21]). In the present study, we investigated the link between inattentional blindness and a multitude of fundamental personality traits. Interestingly, inattentional blindness was largely independent of observers' personality. Basic traits such as extraversion, neuroticism, agreeableness, conscientiousness, BIS, and BAS, as well as a-priori likely candidates such as achievement motivation, schizotypy, and absorption were not related to the likelihood of detecting an unexpected object. However, openness, one of the five most fundamental dimensions of personality, indeed predicted noticing in the present study.

The result that openness is negatively related to inattentional blindness is striking and represents a novel finding both in regard to the concept of openness and, more importantly for the present purpose, in regard to inattentional blindness. We demonstrate that individuals that are open to new experiences in regard to interests, impressions, and ideas are also more "open" to unexpected objects. McCrae ([30], p. 251) states that individuals scoring high on openness hold a more “fluid and permeable structure of consciousness”. This permeability seems to become evident (and somewhat beneficial) at the level of perception: Highly open people can detect stimuli in their environment that do not reach awareness in other people. Conscious perception of individuals scoring high on openness thus seems to be less restricted to expected and task-relevant stimuli, such that unexpected objects can pass the threshold of consciousness more readily. It might be argued that the predictive power of openness is relatively small: Only 1–2% of the variance in inattentional blindness in the present study can be attributed to individual differences in openness to new experiences. It should be noted, however, that this finding is only the second one in the literature on inattentional blindness to establish any relation to a personality trait. It thus corroborates the notion that part of the variability of these failures of awareness is indeed based on stable features of the observer's personality.

Interestingly, our finding on the role of openness is consistent with previous results indicating that inattentional blindness is negatively associated with creative thinking [22]. Openness and creativity are closely linked, although the nature of this relationship is somewhat unclear [30] (see also [60]). Recently, it has been suggested that creative behavior and achievement might be one of two facets of openness to experience, the other one being fluid intelligence [61]. Moreover, creativity is associated with certain styles of attentional processing and perceptual abilities [22,62,63]. Thus, the relation between openness and inattentional blindness, as described in the present study, might actually rely on the following: Open people are characterized by a high level of creativity [60], which entails enhanced perceptual and attentional capabilities [22,62,63], which in turn affects the likelihood of consciously perceiving unexpectedly appearing objects. Clearly, this is a post-hoc explanation that necessitates and warrants further investigation.

It might be argued that the present results regarding the relation between openness and inattentional blindness merely replicate the previous finding that inattentional blindness is negatively related to creativity in children [22]. However, many scholars typically regard creativity and openness as related, yet clearly separable constructs [24,25,64]). This is reflected, for example, by the fact that both measures are typically associated only moderately [65]. In fact, it has even been suggested that extraversion is a better predictor of creativity than openness [25]. Therefore, the present results go beyond previous findings, extending them to a fundamental personality trait and to a large-scale adult sample.

In contrast to Richards et al. [21], we did not find a relationship between inattentional blindness and absorption. The findings of Richards and colleagues seem robust, as they were able to replicate them in a second study. Likewise, the present results might be considered robust due to the extraordinarily large sample on which they are based (*N* = 554). Thus, the discrepancy between the two findings appears to be systematic. We propose that the difference lies in the choice of the specific inattentional blindness task. The tracking task of Richards et al. might pose higher and different demands on attentional capacity than the lexical-decision task in the present study. Individual differences in absorption, the ability to become highly focused and shut out distractors, might be deployed differently in a task that actually contains distractors and demands sustained attention to multiple objects in the display. In fact, Richards et al. demonstrated that the relationship between inattentional blindness and absorption was mediated by working memory capacity. When inattentional blindness occurs during a low-demand task, such as in the present study, working memory presumably does not affect the likelihood of detecting an unexpected object [6].Thus, absorption, which is related to working memory and only contributes to inattentional blindness via this indirect link [21], cannot affect noticing, either. The present results valuably extend previous research on the role of personality in inattentional blindness by suggesting that absorption does not affect inattentional blindness when the cognitive demands of the primary task are low.

Two personality dimensions that might be expected to contribute to inattentional blindness, namely achievement motivation and schizotypy, did not predict such failures of awareness. While achievement motivation showed a borderline significant correlation with inattentional blindness, it was no significant predictor in the regression analysis. This indicates that the influence of motivation on inattentional blindness is limited: In contrast to momentary motivational modulations [37], achievement motivation as a personality trait does not predict inattentional blindness. Assumingly, an overarching drive to succeed does not lead to the employment of additional resources that might be useful in preventing such failures (see [15]). Moreover, individual differences in schizotypy, a person's general proneness to psychosis, were not linked to individual differences in inattentional blindness. Even though schizotypy affects attentional abilities and the processing of irrelevant stimuli [33], this does not seem to generalize to inattentional blindness. Schizotypy leads to less inhibition of present distractors [35], but our results suggest that it does not alter the way in which novel and unexpected events are processed (see [20] for a similar argumentation).

Finally, we explored whether a series of additional fundamental traits were related to inattentional blindness. Our results indicate that extraversion, neuroticism, agreeableness, conscientiousness, BIS, and BAS are not associated with these failures of awareness. We acquired these data in a large sample and achieved strong statistical power, such that it is most unlikely that our results are merely the consequence of insensitive testing. Therefore, inattentional blindness, at least with the paradigm used in the present task, seems to be independent of many features of the observer's personality. This is in line with findings that individual differences in trait negative affect, anhedonic depression, anxious arousal, and worry are not directly linked to inattentional blindness [20].

### Limitations of the Current Study

The fact that we conducted the present study as an online experiment might be considered a limitation of our findings, as online studies can entail diverse and uncontrollable situations during testing. While we could establish that the technical devices used by our participants did not affect their performance, other situational conditions were beyond our reach. This is disadvantageous, as a high variability during testing increases the noise within the data. It should be noted, however, that the high sample size that can be acquired in online experimentation might make up for this detriment. Moreover, we filtered our data carefully and rigorously to eliminate the noise, applying rather strict criteria to select participants for analysis. For example, only participants who performed perfectly on the primary task were included in the analysis to ensure a continuous attentional focus during the task. Also, only participants who noticed the additional object when they were not engaged in a primary task (full-attention trial) were included, which ensures that basic perceptual, technical, and motivational requirements were met by all participants included in the final analysis. And finally, analyses of internal consistency revealed reliabilities of the scales that are both comparable to laboratory testing and sufficiently high to analyze correlations with other measures. This precludes that participants responded in a careless or random way. Nevertheless, the present data (as all experimental data, for that matter) would clearly benefit from replication, in particular in a laboratory setting, even though sample sizes will probably have to be considerably smaller.

In the present study, we included both (a) personality traits that seemed promising candidates for a relationship with inattentional blindness and (b) classical dimensions of personality. This clearly represents only a selection of the vast field of personality dimensions. Thus, there might be other traits predicting inattentional blindness. Additionally, different inattentional blindness paradigms might differ with respect to their link to personality traits. The operationalization of inattentional blindness via a dynamic and sustained task (e.g., [11,27]) might yield different results from ours (which were obtained with a static task; e.g., [1]). The discrepant results in regard to absorption [21] constitute a first hint at such a distinction between paradigms. Future studies are clearly needed to further elucidate this link.

## Conclusion

In a large-scale sample we demonstrated the minimal predictive value of personality in inattentional blindness. We tested the contribution of a variety of personality traits, yet found only openness to be linked to noticing an unexpected object. The effect size was rather small, indicating that this trait accounts only for a fraction of the variability of such failures of awareness. It does, however, corroborate previous studies suggesting that individual differences do play a role in inattentional blindness (e.g., [15,21]).

It might be added that the present study could also be seen as a contribution to central methodological issues in the research on inattentional blindness. Inattentional blindness studies are typically faced with the problem that for each participant only one critical trial can be realized: Once the participant is asked about unexpected events, any subsequent presentation of the additional object is not unexpected any more. This creates the need for large sample sizes. With the present study, we demonstrate that inattentional blindness can be measured as part of an online study (see also [66]), even on a simple survey platform. This might constitute an interesting alternative to laboratory studies and might mitigate the sample-size problem in this area of research.

## Supporting Information

S1 File Additional Analyses. Analyses with different exclusion criteria for participants.
(PDF)
