# Supplementary material for: Some See It, Some Don’t: Exploring the Relation between Inattentional Blindness and Personality Factors
Source: PLoS One. 2015 May 26;10(5):e0128158. doi: 10.1371/journal.pone.0128158 (PMC4443971; doi:10.1371/journal.pone.0128158)
Supplement: S1 File — Analyses with different exclusion criteria for participants. (PDF) [file pone.0128158.s001.pdf]

Additional Analyses

Overview

All of the analyses that are reported in the paper "Some see it, some don't: Exploring the relation between inattentional blindness and personality factors" were performed after carefully excluding participants on the basis of five a-priori criteria:

(A) If participants did not see both inattentional blindness videos because of technical problems.
(B) If participants did not notice the unexpected object in the control condition (full-attention trial)
(C) If participants expected the additional object.
(D) If participants did not perform perfectly on the primary task.
(E) If participants reported impaired vision.

While we believe that all of these points constitute essential exclusion criteria in an online inattentional blindness setting, it might be interesting (e.g., for further online experimentation in this field) to explore whether the selection of criteria altered the general pattern of results. To test this, we re-analyzed all data without excluding any participants, as well as by selectively dropping each of the five criteria.

Results without any exclusions

Of the 1104 participants included in these analyses 72.0% did not notice the unexpected object and, thus, were inattentionally blind. Openness was still significantly, albeit weaker, correlated with noticing (r = .07, p = .03). Achievement motivation was not correlated with noticing (r = .03, n.s.), but gender was (r = .16, p < .001). The regression model that included all a-priori expected predictors was not significant (p = .12) as a whole, but openness constituted the sole significant predictor (p = .01). The regression including openness and gender was not different to the original analysis. None of the other variables were significantly related to inattentional blindness.

Results without excluding participants based on criterion (A)

Of the 586 participants included in these analyses 62.8% did not notice the unexpected object and, thus, were inattentionally blind. Results were nearly identical to those reported in the manuscript: Openness (r = .13, p = .002) and gender (r = .14, p < .001) significantly correlated with noticing. Achievement motivation was weakly correlated with noticing (r = .06, p = .12). The regression model that included all a-priori expected predictors was significant as a whole (p = .007), and openness constituted the sole significant predictor (p = .001). The regression including openness and gender was not different to the original analysis. None of the other variables were significantly related to inattentional blindness.

Results without excluding participants based on criterion (B)

Of the 808 participants included in these analyses 71.7% did not notice the unexpected object and, thus, were inattentionally blind. Results were nearly identical to those reported in the manuscript: Openness (r = .09, p = .01) and gender (r = .16, p < .001) significantly correlated with noticing. Achievement motivation was weakly correlated with noticing (r = .07, p = .04). The regression model that included all a-priori expected predictors was significant as a whole (p = .02), and openness constituted the sole significant predictor (p = .006). The regression including openness and gender was not different to the original analysis. None of the other variables were significantly related to inattentional blindness.

Results without excluding participants based on criterion (C)

Of the 574 participants included in these analyses 62.2% did not notice the unexpected object and, thus, were inattentionally blind. Results were nearly identical to those reported in the manuscript: Openness ($r = .11$, $p = .006$) and gender ($r = .14$, $p = .001$) significantly correlated with noticing. Achievement motivation was weakly correlated with noticing ($r = .09$, $p = .04$). The regression model that included all a-priori expected predictors was significant as a whole ($p = .03$), and openness constituted the sole significant predictor ($p = .02$). The regression including openness and gender was not different to the original analysis. None of the other variables were significantly related to inattentional blindness.

Results without excluding participants based on criterion (D)

Of the 602 participants included in these analyses 63.0% did not notice the unexpected object and, thus, were inattentionally blind. Results were nearly identical to those reported in the manuscript: Openness ($r = .12$, $p = .003$) and gender ($r = .18$, $p < .001$) significantly correlated with noticing. Achievement motivation was weakly correlated with noticing ($r = .09$, $p = .04$). The regression model that included all a-priori expected predictors was significant as a whole ($p = .005$), and openness constituted a highly significant predictor ($p = .002$). On top of that, absorption appeared as a as a significant predictor in this regression as well ($p = .04$). Note, though, that absorption was not at all correlated with noticing ($r = -.01$, $p = .74$). The regression including openness and gender was not different to the original analysis. None of the other variables were significantly related to inattentional blindness.

Results without excluding participants based on criterion (E)

Of the 602 participants included in these analyses 63.0% did not notice the unexpected object and, thus, were inattentionally blind. Results were nearly identical to those reported in the manuscript: Openness ($r = .10$, $p = .02$) and gender ($r = .13$, $p = .001$) significantly correlated with noticing. Achievement motivation was weakly correlated with noticing ($r = .06$, $p = .17$). The regression model that included all a-priori expected predictors was significant as a whole ($p = .045$), and openness constituted the sole significant predictor ($p = .01$). The regression including openness and gender was not different to the original analysis. None of the other variables were significantly related to inattentional blindness.

Summary

In summary, the overall pattern of results stayed the same across all analyses. As could be expected, though, data were noisier, especially if no exclusion criterion was applied. The correlation between achievement motivation and noticing was small in all analyses and fluctuated around the threshold of significance. In no analysis did any of the other variables appear as significant predictors. Openness and gender were robust predictors of inattentional blindness for all variants of exclusion.
